# Incidence and Determinants of Nonfocal Transient Neurologic Attacks: The Rotterdam Study

**DOI:** 10.1212/WNL.0000000000213854

**Published:** 2025-07-08

**Authors:** Bernhard Pieter Berghout, Daniel Bos, Maryam Kavousi, Mohammad Arfan Ikram, Mohammad Kamran Ikram

**Affiliations:** Department of Epidemiology, Erasmus MC - University Medical Center, Rotterdam, the Netherlands.

## Abstract

**Background and Objectives:**

Recent literature suggests no elevated risk of cardiovascular disease in patients having nonfocal transient neurologic attacks (TNAs), yet the origin of these attacks remains unclear. Therefore, we investigated their incidence and potential risk factors in a prospective cohort study, hypothesizing associations with cardiovascular risk factors.

**Methods:**

Within the Dutch population–based Rotterdam Study, community-living individuals aged 45 years or older underwent assessment for demographic and cardiovascular risk factors in study cohorts initiated in 1990, 2000, and 2006. Participants were subsequently followed until January 1, 2021, for disease incidence through automated linkage of the study database with medical records from participants' general practitioners. For this study, participants free from TNA at baseline were selected and followed up for the outcome of a nonfocal TNA. The risk of nonfocal TNA was determined through age-specific incidence rates (IRs). Potential risk factors of nonfocal TNA were identified using cause-specific, multivariable, Cox proportional hazard modeling accounting for age, sex, education (university or higher vocational vs lower education level), cardiovascular risk factors, ultrasound markers of carotid atherosclerosis, antithrombotic medication use, and a history of vascular diseases. Sensitivity analyses consisted of conducting this regression among participants without any history of vascular disease at study entrance.

**Results:**

After 204,474 person-years of follow-up in 14,096 participants (mean [SD] age 65.5 [10.3] years, 59.0% female), 518 index nonfocal TNAs (3.7%) occurred. The incidence of nonfocal TNA increased with age, with an IR in those aged between 55 and 59 years of 65.5 (95% CI 33.6–116.2) per 100,000 person-years of follow-up to 424.0 (95% CI 348.0–512.0) in those aged 85 years or older. Older age (hazard ratio [HR] 1.08, 95% CI 1.07–1.09), a lower education level (HR 1.62, 95% CI 1.15–2.29), plaque presence on carotid ultrasound (HR 0.80, 95% CI 0.65–1.00), and the use of antithrombotics (HR 0.59, 95% CI 0.39–0.90) were all independently associated with the risk of nonfocal TNA. After restricting analyses to the 12,499 individuals without history of vascular disease, only the associations of age and a higher maximum attained education level with the risk of nonfocal TNA remained statistically significant.

**Discussion:**

Nonfocal TNAs predominantly affect older adults and those with lower educational attainment, suggesting that these attacks originate from socioeconomic determinants rather than from established cardiovascular risk factors.

## Introduction

Transient neurologic attacks (TNAs) are sudden episodes of neurologic symptoms lasting <24 hours without a clear alternative diagnosis.^1^ These attacks present in focal, nonfocal, and mixed forms, based on the nature of the symptomatology. Focal and mixed TNAs are generally regarded as typical and atypical TIAs while nonfocal TNAs are poorly understood and are often considered in clinical settings as more benign occurrences when compared with their counterparts with focal deficit.^2^

These notions seem to be confirmed by recent evidence suggesting that individuals who experience nonfocal TNA do not have an elevated subsequent risk of cardiovascular diseases,^3^ although this finding contrasts with older studies.^1,4^ Furthermore, previous cross-sectional studies have associated nonfocal TNAs with markers of cerebral small vessel disease,^5^ MRI-detected acute ischemia,^6^ and carotid artery occlusions.^7^ These associations imply a possible vascular origin for these attacks, regardless of whether they have cardiovascular consequences that necessitate the use of secondary preventative measures.

To elucidate the nature of these nonfocal TNAs, we investigated the overall risk and possible risk factors of these attacks in a Dutch population–based cohort study of middle-aged people. We hypothesized that nonfocal TNAs have a cardiovascular basis, anticipating associations with established cardiovascular risk factors such as hypertension, diabetes mellitus, and dyslipidemia.

## Methods

### Study Setting

This study is embedded in the Rotterdam Study, a population-based, observational cohort study among residents of the Ommoord district in Rotterdam, the Netherlands. The Rotterdam Study was initiated in 1990 to assess demographic changes leading to an increasing proportion of older people in western countries, as well as to investigate factors and trends associated with aging-related disorders. Extensive details regarding the study's rationale and design are described elsewhere.^8^ In brief, all study participants underwent initial examination at the study research center and are invited for follow-up visits every 3–6 years. Meanwhile, their medical records at general practitioners' offices are continuously monitored through electronic linkage with the study database. General practitioners are pivotal to the Dutch health care system, acting as gatekeepers to further specialized medical care and overseeing residents' health status by coordinating hospital discharge notes, imaging summaries, and paramedical reports. This continuous monitoring of health records thus allows for the detection of clinical end points of interest.

### Assessment of Potential Determinants of Nonfocal TNA

For this study, we assessed the effects of baseline demographic and cardiovascular determinants on the risk of TNA. Age at baseline, sex, medication use, smoking habits, and highest attained educational status were assessed through self-report in the initial home interviews, after which we further dichotomized education status (“high,” i.e., university or higher vocational vs lower education level) and smoking status (current or not current smoking). Subsequently, patients were invited for blood sampling, examination, and measurements at the research center. In this study, we calculated body mass index using the measured weight in kilogram, divided by the measured height in meters of participants. Blood pressure was measured at the right brachial artery with the participant in sitting position. The mean of 2 consecutive measurements was used in analyses. Drugs categorized by the World Health Organization Anatomical Therapeutic Chemical classification^9^ as antihypertensives (c02), diuretics (c03), beta blockers (c07), calcium channel blockers (c08), and RAAS-modifying agents (c09) were considered as blood pressure–lowering medication. Hypertension was defined as a resting blood pressure exceeding 140/90 mm Hg or the use of blood pressure–lowering medication. Type 2 diabetes mellitus was defined by either a serum nonfasting glucose level ≥11.1 mmol/L, having a fasting glucose level ≥7.0 mmol/L, or the use of any diabetic medication. Dyslipidemia was defined by either a serum total cholesterol level ≥6.2 mmol/L, a non-HDL cholesterol level ≥5.6 mmol/L, or the use of lipid control medication. Participants also underwent longitudinal 2D ultrasound examination of both common carotid arteries to establish the presence of carotid atherosclerotic disease (CAD). Presence of plaque on carotid ultrasound and mean carotid intima-media thickness (IMT) were used as markers of CAD.^10^ Prevalent atrial fibrillation, coronary heart disease defined by either acute myocardial infarction or elective percutaneous coronary intervention or coronary artery bypass grafting, and stroke were assessed in the interview and checked with medical records in general practitioner files.^11,12^

### Assessment of TNA

Incident TNAs were identified as part of the larger stroke follow-up using medical documents from the general practitioner records linked with the study database, which has been described in detail elsewhere.^3,11,13^ Trained research personnel screened medical documents originating from general practitioner visit notes, emergency department discharge letters, hospital discharge letters, or letters from nursing home physicians for any mention of symptoms related to our definition of a TNA. We defined TNA as an attack of sudden neurologic symptoms that completely resolved within 24 hours, with no clear evidence for the diagnosis of migraine, epilepsy, Menière disease, hyperventilation, cardiac syncope, hypoglycemia, or orthostatic hypotension. Records of potential TNA events were reviewed for content and these alternative diagnoses by research physicians and subsequently verified in consensus with experienced neurovascular specialists to adjudicate TNA events. This process is known to enhance the accuracy of detecting clinical events through record-based assessments.^14^ If only focal neurologic deficit was reported, that is, limb weakness, loss of sensation, aphasia, anopsia, dysarthria, dysphagia, ataxia, diplopia, or vertigo, then the event was classified as a focal TNA. If only nonfocal symptoms were reported, that is, decreased consciousness, unconsciousness, amnesia, confusion, positive visual phenomena, unsteadiness, nonrotatory dizziness, paresthesias, or bilateral weakness, then the event was classified as a nonfocal TNA. This list of nonfocal symptoms was defined as broadly as possible to limit prejudgments from research physicians in detecting possible events. When both focal and nonfocal symptoms were reported, then the event was classified as a mixed TNA. Both focal and mixed TNAs were considered TIA. A previous reproducibility study, where the same neurologist reclassified 121 case histories after being blinded to the initial diagnosis, revealed a weighted Kappa of 0.77, indicating good reproducibility of this method.^13^

### Statistical Analyses

Summary characteristics of participants at baseline are given using frequencies and percentages, or mean and standard deviation for continuous variables. Next, we assessed the rates of index nonfocal TNA in the study by calculating incidence rates (IRs) per 100,000 person-years, in addition to 95% CIs using Byar approximation. We then stratified these IRs by age using 5-year categories starting at ≥55 years. In this method, participants contribute follow-up time to each successive 5-year age category they belong to as they age during study follow-up. We further standardized the overall IR according to the 2010 estimated European Standard Population.^15^

Next, potential risk factors of nonfocal TNAs were determined by examining associations between baseline characteristics and the incidence of nonfocal TNA. To this end, hazard ratios (HRs) and 95% CIs were obtained from a multivariable cause-specific hazard model, treating all-cause mortality as a competing risk. Next, we repeated this analysis among study participants without prevalent CHD, stroke, and atrial fibrillation, to detect potential risk factors among individuals without a history of vascular disease. As a sensitivity analysis, we applied the initial regression model while accounting for the other TNA types as additional competing risks. In this manner, participants were censored at the occurrence of any index TNA or at all-cause mortality. We conducted this analysis using TIA as a positive control to the analyses on only nonfocal TNA risk, anticipating that traditional cardiovascular risk factors are significant determinants of the risk of TIA.

All analyses were performed in R version 4.2.2, using the packages “mice” 3.16.0, “survival” 3.5.7, and “cmprsk” 2.2.11. Missing values among covariates were imputed using the mice algorithm, with 30 iterations and 30 imputations, and using data on the included determinants and outcome status as predictors of missing data. We had <11.0% missing data for all predictors, except for serum glucose (13.4%), plaque presence (20.3%), and IMT (21.6%).

### Standard Protocol Approvals, Registrations, and Patient Consents

This work follows the STROBE reporting guideline for observational studies. The Rotterdam Study has been approved by the Medical Ethics Committee of the Erasmus MC (registration number MEC 02.1015) and by the Dutch Ministry of Health, Welfare and Sport (Population Screening Act WBO, license number 1071272-159521-PG). The Rotterdam Study Personal Registration Data collection is filed with the Erasmus MC Data Protection Officer under registration number EMC1712001. The Rotterdam Study has been entered into the Netherlands National Trial Register and into the WHO International Clinical Trials Registry Platform under shared catalogue number NTR6831. All participants provided written informed consent for participation in the study and for researchers to access medical information from their personal physicians.

### Data Availability

The data underlying this article will be shared on reasonable request to the corresponding author. All requests are directed toward the management team of the Rotterdam Study (secretariat.epi@erasmusmc.nl), which has a protocol for approving data requests. Because of restrictions based on privacy regulations and informed consent of the participants, the data underlying this article cannot be made freely available in a public repository.

## Results

### Sample Size and Characteristics

At baseline, 14,926 participants were enrolled into the study. Of these, 391 participants (2.6%) have retracted informed consent for the use of follow-up information over the course of the study and were thus excluded from this selection. Of the remaining 14,535 participants, 439 (2.9%) had a history of any TNA before study enrollment and were subsequently removed from the final analysis selection because they were not at risk of an index TNA during follow-up. The remaining 14,096 participants were followed up from baseline, until a combined censoring point of either index nonfocal TNA, death, last health status update when they were known to be free of TNA, or the end of the study period on January 1, 2021, whichever came first. Follow-up in this manner was complete for 99.6% of potential person-years of follow-up, with a total follow-up duration of 204,474 (median 13.9, interquartile range 9.6–20.0) person-years. Table 1 summarizes the baseline characteristics of the study participants. At the start of the study, participants were on average 65.6 (SD 10.3) years old and 8,364 (59.0%) were female. During follow-up, 521 people (3.7%) experienced an index nonfocal TNA.

**Table 1:** Baseline Characteristics of Study Participants

Characteristic	N = 14,096
Age at baseline, y, mean (SD)	65.5 (10.3)
Female sex, n (%)	8,364 (59.0)
Education level, n (%)	
Primary	2,541 (18.0)
Low	5,505 (39.1)
Medium	3,712 (26.3)
High	2,058 (14.6)
BMI, kg/m^2^, mean (SD)	26.9 (4.1)
Obesity (BMI ≥30 kg/m^2^), n (%)	2,410 (17.1)
Smoking behavior, n (%)	
Never	4,582 (32.5)
Former	5,884 (41.7)
Current	3,357 (23.8)
Systolic blood pressure, mm Hg, mean (SD)	138 (21.6)
Diastolic blood pressure, mm Hg, mean (SD)	77.3 (12.0)
Blood pressure medication use, n (%)	4,269 (30.3)
Hypertension, n (%)	8,018 (56.9)
Diabetes mellitus, n (%)	1,176 (8.3)
Serum total cholesterol, mmol/L, mean (SD)	6.2 (1.2)
Serum HDL cholesterol, mmol/L, median (IQR)	1.3 (1.1–1.6)
Serum non-HDL cholesterol, mmol/L, mean (SD)	4.8 (1.3)
Lipid-lowering medication use, n (%)	1,332 (9.4)
Dyslipidemia, n (%)	6,830 (48.5)
Mean IMT, per 0.1 mm, median (IQR)	8.0 (7.2–9.0)
Presence of plaque on CUS, n (%)	7,410 (52.6)
Antithrombotic medication use, n (%)	1,220 (8.7)
Prevalent atrial fibrillation, n (%)	540 (3.8)
Prevalent CHD, n (%)	892 (6.3)
Prevalent stroke, n (%)	377 (2.7)

Abbreviations: BMI = body mass index; CHD = coronary heart disease; CUS = carotid ultrasound; HDL = high-density lipid; IMT = intima-media thickness; IQR = interquartile range; n = frequency.

Missing data were present in education (2.0%), BMI (10.5%), smoking (1.9%), blood pressure (9.7%), glucose (13.4%), total cholesterol (10.6%), HDL cholesterol (10.8%), medication use (0.4%), carotid plaque (20.3%), IMT (21.6%).

### Risk of Nonfocal TNA

The overall IR of index nonfocal TNA was 253.3 (95% CI 232.2–275.9) per 100,000 person-years of follow-up. This did not significantly vary by sex, with men having an IR of 231.2 (95% CI 199.9–266.2) per 100,000 person-years of follow-up and women having an IR of 267.8 (95% CI 240.1–297.8) per 100,000 person-years of follow-up. Table 2 summarizes the age-specific IRs of nonfocal TNA, depicting an increase in incidence by older age.

**Table 2:** Age-Specific IRs of Index Nonfocal TNA in the Rotterdam Study

Age category	No. of cases	No. of person-years of follow-up	IR per 100,000 person-years	95% CI
<55	0	6,260.0	—^a^	—
55–59	10	15,268.0	65.5	34.6–116.2
60–64	32	30,634.3	104.5	72.8–145.6
65–69	64	35,285.5	181.4	140.9–230.0
70–74	106	35,314.7	300.2	247.0–361.5
75–79	100	32,004.0	312.5	255.6–378.3
80–84	103	25,416.7	405.3	332.6–489.4
85+	103	24,290.8	424.0	348.0–512.0
Total	518	204,474.0	253.3	232.2–275.9
Age-standardized	—	—	214.8^b^	168.4–270.2

Abbreviations: IR = incidence rate; TNA = transient neurologic attack.

a IRs were omitted because of no incident cases within this age group.

^b^Standardized to the 2010 European Standard Population.

### Risk Factors of Nonfocal TNA

Table 3 summarizes the results of multivariable cause-specific hazard regression analyses assessing the effect of demographic and cardiovascular characteristics on the risk of nonfocal TNA. Nonfocal TNA occurred more frequently among participants of older age (HR per 5-year increase 1.47, 95% CI 1.40–1.55) and with lower education (HR 1.62, 95% CI 1.15–2.29). Nonfocal TNA occurred less frequently among participants using any antithrombotic medication (HR 0.59, 95% CI 0.39–0.90) and with atherosclerotic plaque on their carotid ultrasound (HR 0.80, 95% CI 0.65–1.00). These effect estimates did not significantly change after repeating the analyses in the sample without prevalent vascular diseases such as CHD, stroke, or atrial fibrillation, although the associations of the use of any antithrombotic medication and carotid plaque presence with the risk of nonfocal TNA were no longer statistically significant.

**Table 3:** Results of Cause-Specific Hazard Regression Analyses on the Risk of Index Nonfocal TNA

Frequency (n/N)	Nonfocal TNA, full study (518/14,096)		Nonfocal TNA, no prevalent CVD (460/12,449)	
Determinants	*n* (%)	HR (95% CI)	*n* (%)	HR (95% CI)
Age, per 5 y	—	1.47 (1.40–1.55)	—	1.49 (1.41–1.58)
Sex, female	331 (63.9)	0.88 (0.72–1.07)	299 (65.0)	0.83 (0.68–1.02)
Lower education	472 (91.1)	1.62 (1.15–2.29)	415 (90.2)	1.49 (1.05–2.12)
Obesity	86 (16.6)	0.94 (0.74–1.19)	80 (17.4)	1.01 (0.78–1.30)
Current smoking	96 (18.5)	1.01 (0.80–1.27)	84 (18.3)	0.99 (0.77–1.26)
Hypertension	327 (63.1)	1.15 (0.95–1.39)	280 (60.9)	1.13 (0.93–1.38)
Diabetes mellitus	37 (7.1)	0.92 (0.65–1.29)	32 (7.0)	0.99 (0.68–1.42)
Dyslipidemia	276 (53.3)	1.13 (0.95–1.35)	241 (52.4)	1.12 (0.93–1.35)
Plaques on CUS	254 (49.0)	0.80 (0.65–1.00)	221 (48.0)	0.83 (0.66–1.04)
Greater IMT on CUS, per 0.1 mm	—	0.99 (0.92–1.07)	—	0.99 (0.91–1.08)
Antithrombotic medication use	28 (5.4)	0.59 (0.39–0.90)	13 (2.8)	0.59 (0.34–1.03)
Prevalent atrial fibrillation	13 (2.5)	0.60 (0.34–1.05)	—	—
Prevalent CHD	30 (5.8)	1.00 (0.68–1.47)	—	—
Prevalent stroke	17 (3.3)	1.48 (0.90–2.44)	—	—

Abbreviations: CHD = coronary heart disease; CUS = carotid ultrasound; CVD = cardiovascular disease; IMT = intima-media thickness; N = total; n = events; *n* = number and prevalence of cases with determinant; TNA = transient neurologic attack.

### Sensitivity Analyses With Censoring for Index Any TNA, Including Both TIA and Nonfocal

Estimates on the risk and risk factors of index any TNA, stratified by nonfocal TNA and TIA, are presented in eTable 1. One in 9 individuals experienced any TNA, with a total of 1,586 index TNAs (11.3%) occurring. Of these, 1,121 (70.7%) were TIAs and 465 (29.3%) were nonfocal in nature. No notable changes in the effect estimates of risk factors of nonfocal TNA occurred in this sensitivity analysis. Expectedly, cardiovascular risk factors were associated with the risk of TIA, as seen by the effects in participants of older age (HR per 5-year increase 1.30, 95% CI 1.26–1.35) and those with hypertension (HR 1.22, 95% CI 1.07–1.40), dyslipidemia (HR 1.13, 95% CI 1.00–1.27), increased IMT on their carotid ultrasound (HR 1.06, 95% CI 1.00–1.12), and prevalent stroke (HR 1.91, 95% CI 1.40–2.63).

## Discussion

In this population-based study of Dutch middle-aged and older individuals, we observed that approximately 1 in 9 people will develop a TNA during their lifetime, with almost one-third of these attacks being nonfocal in nature. Notably, none of the classical cardiovascular risk factors were associated with an increased risk of nonfocal TNA. Instead, nonfocal TNAs were more frequently observed among older participants and those with a lower maximum attained education level.

Comparing IRs of TNA with those of other countries is challenging because of a lack of comparable data in the literature. However, some extrapolation is possible when assessing the IRs for TIA. Notably, we report a crude IR of TIA that is almost 5 times higher than that observed in the comparable Framingham Heart Study.^16^ This suggests that our population may experience TNA more frequently, although this difference could also be attributed to a higher sociodemographic status or ethnic differences between our study population and that of the Framingham Heart Study.

We found that no classical cardiovascular risk factor was independently associated with the risk of nonfocal TNA, which contrasts with previous studies. Specifically, occluded internal carotid arteries,^7^ MRI markers of cerebral small vessel disease,^5^ and cerebral hypoperfusion^6^ have been suggested as potential causes for nonfocal TNAs, all known entities associated with classical cardiovascular risk factors such as hypertension and diabetes.^17^ The latter of these proposed causes has particularly been suggested to be responsible for nonfocal TNAs resembling dysfunction in the vertebrobasilar territory, such as nonrotatory dizziness and bilateral weakness.^18-20^ Nevertheless, we did not find any evidence for any association with cardiovascular risk factors in this study, suggesting that nonfocal TNAs occur for a different reason than a cardiovascular disturbance on a population level. Possible reasons for our contrasting results may be attributed to differences in study design and underlying study population. Previous studies are predominantly cross-sectional in nature, which are less suitable for establishing temporal relationships when compared with this study with extensive follow-up. Furthermore, these studies were performed in hospital settings, which restrict analyses to patient samples only, possibly leading to overestimations of effects.

Paradoxically, while our findings suggest that nonfocal TNAs have no cardiovascular basis, we also observed that individuals with carotid plaques and those using antithrombotic medication were at decreased risk of nonfocal TNA. However, these effects attenuated and lost statistical significance after excluding individuals with a history of cardiovascular disease. Alternatively, the sample size reduction induced by excluding individuals with a history of cardiovascular disease may have led to a reduced ability to detect a statistically significant association between these facets and the risk of nonfocal TNA.

Rather than the previously suggested cardiovascular etiology behind nonfocal TNA, our findings suggest a potential socioeconomic one instead. Specifically, a lower education level emerged as an independent determinant of a higher risk of nonfocal TNA. Individuals with lower education may have a less favorable environment with difficult access to health care, worse compliance to treatments with a subsequent increase in a variety of side effects, an unhealthier lifestyle in general, or a lower level of health literacy.^21,22^ Each of these aspects may contribute to an increased incidence of nonfocal TNAs among individuals with a lower level of education when compared with individuals with a higher level of education, or at the least, make them more inclined to seek medical attention for these attacks. Given the higher incidence of nonfocal TNAs among less educated individuals, a more in-depth diagnostic workup may be warranted in these patients to determine whether their symptoms are truly nonfocal rather than a misdiagnosed TIA.^6^ This is particularly important in light of recent evidence showing substantial differences in the risk of cardiovascular disease between TIA and nonfocal TNA, underscoring the need for secondary prevention in the former but not at all for the latter.^3^

Strengths of this study include its population-based, prospective design with over 30 years of follow-up data from a large cohort of older participants. In addition, our method of scrutinizing medical records and subsequently reviewing them in a consensus-based evaluation with several vascular experts is another strength. This method is reported to increase diagnostic accuracy,^14^ ensuring valid detection of possible TNA events. These strengths enabled us to precisely assess the long-term risk and risk factors of nonfocal TNAs.

However, several key limitations of this study must be acknowledged. First, our medical record–based detection of possible TNA events precluded personal in-depth interviews with patients, which could have provided a more complete account of their attack in addition to the time elapsed between symptom onset and medical documentation. However, such an extensive method is impractical for a population-level investigation, and the present method likely remains the most effective option for population-based studies. Despite this, some participants may have been misclassified as TNA-free individuals because of our reliance on medical records, as not everyone suffering a TNA will seek medical attention. This is evidenced by reports indicating that 1 in 5 stroke patients had TIA symptoms and did not seek medical attention,^23^ suggesting potential underreporting in our study. In addition, while the general practitioner records allow for an accurate assessment of the medical history of participants at study entrance, we were unable to cross-reference the assessment of determinants that were strictly assessed in personal interviews only (i.e., education status and smoking), which may have led to misclassification bias for these determinants.

Second, the lack of any association between classical cardiovascular risk factors and the risk of nonfocal TNA could also be explained by insufficient power to detect these associations. Specifically, for hypertension and dyslipidemia, the estimates for nonfocal TNA have a similar directional effect to those for TIA, but the 95% CIs indicate that these effects for nonfocal TNA are not statistically significant. A larger sample size might reveal a statistically significant association between these risk factors and the risk of nonfocal TNA, although the magnitude of the effects found indicates that the clinical significance of such a finding could be questionable. Furthermore, this study has included a substantial number of nonfocal TNA cases followed prospectively over 30 years, yet the advanced age and increasing mortality of participants limit the potential to accrue additional nonfocal TNA cases and subsequently increase statistical power.

Finally, the generalizability of these findings is limited by the primarily Western European background of the Rotterdam Study participants. This complicates extrapolating our findings to populations with different socioeconomic or ethnic backgrounds. Future estimates on the risk of nonfocal TNA from more diverse populations could help to put these results in a broader context.

In conclusion, the risk of nonfocal TNA is elevated in older and less educated individuals but does not seem to be associated with cardiovascular risk factors. These findings improve our understanding of the origin of these attacks, suggesting that they are not due to a cardiovascular pathophysiology requiring cardiovascular risk management or secondary prevention akin to that for a TIA. This may in turn aid clinical decision making and provide context for patients experiencing nonfocal TNAs.

## Glossary

CAD: carotid atherosclerotic disease
HR: hazard ratio
IMT: intima-media thickness
IR: incidence rate
TNA: transient neurologic attack
